# Abnormal movements in critical care patients with brain injury: a diagnostic approach

**DOI:** 10.1186/s13054-016-1236-2

**Published:** 2016-03-14

**Authors:** Yousef Hannawi, Michael S. Abers, Romergryko G. Geocadin, Marek A. Mirski

**Affiliations:** Neurosciences Critical Care Division, Department of Anesthesiology and Critical Care Medicine, Johns Hopkins University, Baltimore, MD USA; Department of Neurology, Johns Hopkins University, Baltimore, MD USA; Present address: Division of Cerebrovascular Diseases and Neurocritical Care, Department of Neurology, The Ohio State University, Columbus, OH USA; Department of Medicine, Massachusetts General Hospital, Harvard Medical School, Boston, MA USA; Department of Neurosurgery, Johns Hopkins University, Baltimore, MD USA

## Abstract

Abnormal movements are frequently encountered in patients with brain injury hospitalized in intensive care units (ICUs), yet characterization of these movements and their underlying pathophysiology is difficult due to the comatose or uncooperative state of the patient. In addition, the available diagnostic approaches are largely derived from outpatients with neurodegenerative or developmental disorders frequently encountered in the outpatient setting, thereby limiting the applicability to inpatients with acute brain injuries. Thus, we reviewed the available literature regarding abnormal movements encountered in acutely ill patients with brain injuries. We classified the brain injury into the following categories: anoxic, vascular, infectious, inflammatory, traumatic, toxic-metabolic, tumor-related and seizures. Then, we identified the abnormal movements seen in each category as well as their epidemiologic, semiologic and clinicopathologic correlates. We propose a practical paradigm that can be applied at the bedside for diagnosing abnormal movements in the ICU. This model seeks to classify observed abnormal movements in light of various patient-specific factors. It begins with classifying the patient’s level of consciousness. Then, it integrates the frequency and type of each movement with the availability of ancillary diagnostic tests and the specific etiology of brain injury.

## Background

An important, yet understudied, area of critical care medicine is the diagnostic approach to abnormal movements in patients with brain injuries. Abnormal movements, alternatively described as dyskinesias or “paroxysmal” motor phenomena, often serve as valuable clues to the etiology of the injury and they have the potential to help guide treatment and prognostication [1]. In addition, early recognition of the abnormal movement may avoid unnecessary and often expensive diagnostic evaluation [2].

Seizure as a cause of motor paroxysms in the intensive care unit (ICU) cannot be overstated, as early recognition and treatment may improve patients’ outcomes [3]. However, the observation that “not every shake in the ICU is a seizure” must also be emphasized. In a small study of 52 patients who underwent video-electroencephalogram (EEG) testing for suspicion of seizures in the ICU, epileptic seizures were identified as the etiology in only 27 % of all the events; the remainder were attributed to other etiologies, including tremor, multifocal myoclonus or semi-purposeful movements [4]. Thus, there is a need for a systematic diagnostic approach to differentiating these movements.

**Fig. 1:**
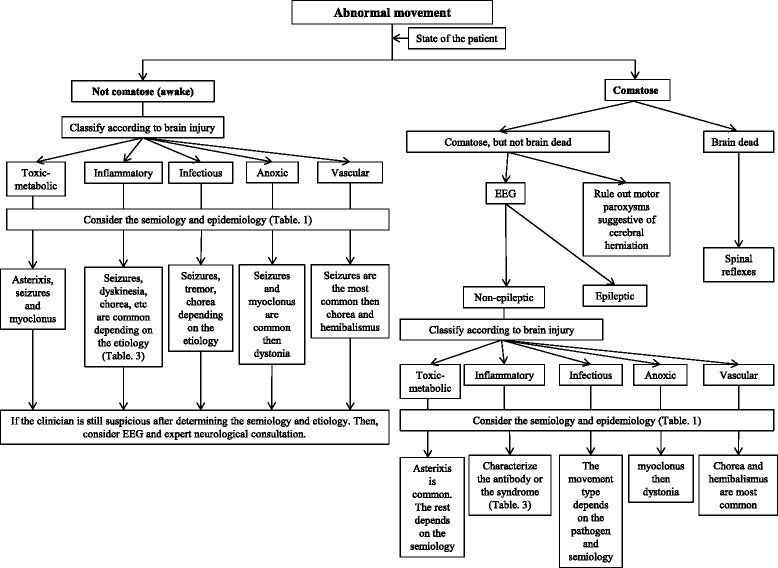
A diagnostic approach to abnormal movements in patients with brain injury. We propose an approach based on the mental state first. It is important to rule out seizures first in comatose patients. Knowledge of the etiology and underlying brain damage may provide valuable information for differentiating the various types of abnormal movements. *EEG* electroencephalogram

The typical and logical diagnostic approach to dyskinesia in the neurological subspecialty field of movement disorders is based on establishing the phenomenology, which leads to categorizing the movement and eventually generating a differential diagnosis of the possible etiologies [5]. The inter-observer reliability of such an approach is dependent on the operator expertise and such reliability has not been evaluated among intensivists [6–8]. In addition, expert neurologists may not be readily available in the ICU. Moreover, it is difficult to implement such an approach even by expert neurologists in certain ICU patients with brain injury since they are often comatose or uncooperative, limiting the clinician’s ability to conduct a detailed neurological assessment. Furthermore, the etiology is often known at the time of ICU admission, limiting the usefulness of this approach. Thus, to create a practical paradigm for approaching abnormal movements in brain injury patients that takes into account the special case of ICU patients, we performed a selective review of the literature available through PubMed using the keywords “abnormal movement”, “movement disorders”, “dystonia”, “tremor”, “chorea”, “myoclonus”, “seizures”, “brain injury”, “stroke”, “subarachnoid hemorrhage”, “traumatic brain injury”, “brain tumor”, “cardiac arrest”, and “paraneoplastic syndromes”. We propose an approach based on the integration of the abnormal movement epidemiology, semiology, etiology of brain injury and mental state of brain injury patients. This approach is based first on classifying the patient’s level of consciousness as comatose or awake. In the comatose state, identification of brain death state shifts the diagnostic paradigm towards spinal reflexes and avoids unnecessary work-up. Otherwise, comatose patients should undergo an EEG or continuous EEG (cEEG) to rule out seizures or non-convulsive status epilepticus (NCSE). Following these steps, an approach based on the integration of semiology and epidemiology according to the brain injury type will likely yield the correct diagnosis (Fig. 1).

A different approach may be taken in awake patients. Careful attention to the phenomenology and epidemiology of the abnormal movement according to the brain injury may be the initial step (Fig. 1). An EEG may be used as an ancillary test in difficult cases or when the category of brain injury is highly epileptogenic. Expert neurological consultation can be sought in difficult cases. In the following sections, we review the basic definitions and clinical characteristics of abnormal movements encountered in the ICU. Then, we review these abnormal movements according to the type of brain injury.

## Definitions

There are two principal categories of neurological syndromes in the field of movement disorders: those with a paucity of voluntary or automatic movements (hypokinesia) and those with excessive unnatural movements (interchangeably referred to as hyperkinesia, dyskinesia, or abnormal involuntary movements) [5]. Seizures, paroxysmal posturing and spinal reflexes are also well known to produce motor findings in the ICU [9, 10]. For the purpose of this review and to create a practical approach for ICU patients, the term “abnormal movements” will refer to the variety of movements seen in the ICU accompanying different types of brain injuries, including the motor findings of seizures and NCSE, paroxysmal posturing movements accompanying cerebral herniation, shivering, clonus and the five major categories of dyskinesia in the field of movement disorders (chorea, dystonia, myoclonus, tics and tremor) [5, 9–11] (Table 1).

**Table 1:** Abnormal movement categories and their pathological origin listed in alphabetical order

Abnormal movement	Definition	Pathophysiological origin
Chorea	Involuntary, purposeless, nonrhythmic, non-sustained movements that flow from one body part to the other	Poorly understood. Could be due to loss of normal pallidal inhibitory input
	Hemiballismus: a severe form of chorea, is characterized by vigorous irregular high amplitude movements on one side of the body	Hemiballismus happens secondary to injury of the subthalamic nucleus
Clonus	Rhythmic involuntary muscular contractions and relaxations	Upper motor neuron injury and its descending pathways
Dystonia	Sustained twisting movements that are often frequent and progresses to prolonged abnormal postures	Basal ganglia. Abnormalities are also seen in the cortex and reduction in spinal cord and brainstem inhibition
Myoclonus	Sudden, brief involuntary movements which may be caused by muscle contractions (positive myoclonus)	Widespread origin depending on the injury or type: cortical, subcortical (basal ganglia), brainstem or spinal cord in segmental myoclonus
	Asterixis is considered a negative myoclonus secondary to sudden loss of tone	
NCSE	Unilateral eye deviation, lip smacking, automatisms and some movements of the fingers	Cortical in origin
Paroxysmal posturing	Involuntary flexor or extensor posturing on one side or bilateral spontaneously or with pain. Opisthotonus posturing refers to hyperextension of the neck and back “arching position”	Damage above the red nucleus (flexion posturing) or below (extensor posturing) Midbrain injury or tetanus (opisthotonus)
Shivering	High frequency involuntary muscular contractions involving one group or more of muscles	Thermoregulatory (due to hypothermia) or non-thermoregulatory (not well understood)
Tics	Abnormal movements (motor) or sounds (phonic) which can be simple muscle jerks or complex when they consist of sequential movements in different parts of the body	May be related to abnormalities in the basal ganglia
Tremor	Oscillatory rhythmic movement that affects one or more parts of the body	Likely related to the presence of central oscillator in the basal ganglia or cerebellum

Description and pathophysiology of various categories of abnormal movements that may be seen in intensive care unit patients. *NCSE* non-convulsive status epilepticus

Hypokinesias are frequently seen in outpatient settings and, with the exception of catatonic psychosis, are rarely included within a differential diagnosis of coma [12]. On occasion, however, hypokinetic movements may accompany life threatening emergencies, such as rigidity in neuroleptic malignant syndrome [13]. Hyperkinetic movements (Table 1) and seizures are the most likely paroxysmal movements observed in the ICU setting [14–16]. Some features may help in differentiating these various types of movements. For example, motor phenomena of seizures are often associated with changes in vital signs such as tachycardia, hypertension or brief hypoxia as opposed to tremor, chorea or shivering [17]. Taking into consideration the distribution, frequency and duration of the movement may help as well. Chorea may be unilateral when it results from stroke while shivering tends to be bilateral or involves a muscular group [17], tremor often lasts longer than seizure twitches, higher frequency twitches suggest shivering more than seizures [18], and paroxysmal posturing due to cerebral herniation can be provoked by noxious stimuli compared with seizures, which tend to be spontaneous. Recently, however, stimulus-induced seizures have also been described [19].

## Abnormal movements in comatose patients

Coma is a state of unresponsiveness due to various types of severe brain insults [20]. Different types of abnormal movements may be seen in the comatose state and may represent motor paroxysms in the setting of cerebral herniation, such as flexor or extensor posturing secondary to severe brain injury and subsequent cerebral edema (Table 1) [20]. On the other hand, the abnormal movement may be a direct consequence of the underlying primary insult (discussed below for each category of brain injury). Moreover, epileptic phenomena producing subtle motor activity is also common in the comatose state [21]. Thus, an approach based on recognition of seizures and signs of cerebral herniation and elevated intracranial pressure should initially be undertaken, as urgent medical or surgical intervention may alter the outcome of these patients [20]. After ruling out these etiologies, integrating the semiology and epidemiology of various abnormal movements according to the brain injury type may lead to the correct diagnosis, as discussed for each category of brain injury (Fig. 1). Moreover, the special state of brain death as a sequela of devastating injury needs to be recognized early as it dramatically changes the diagnostic approach (Fig. 1).

### Brain death

Brain death is defined as the irreversible cessation of all brain and brain stem function secondary to catastrophic brain injury [22]. Despite brain inactivity, such patients are not always still and often exhibit a number of spinal reflex movements that may be complex [23]. The diagnosis can be often made clinically. Establishing the diagnosis may avoid unnecessary diagnostic testing. We believe that ruling out brain death should be the first step in approaching abnormal movement in an unresponsive patient (Fig. 1).

The most common movements observed in brain death include flexor or extensor plantar response, triple flexion, abdominal reflex, cremasteric reflex, tonic neck reflexes, and isolated jerks of the upper extremities [23]. Rare complex movements have been observed in the literature, such as repetitive leg movements resembling sleep periodic leg movements [24] and “Lazarus sign”, described as rapid arm flexion with shoulder adduction, followed by returning of the arms to the patient’s side, sometimes asymmetrically [25].

## Abnormal movements according to the type of brain injury

### Convulsive seizures and NCSE

Seizures and status epilepticus may be the sole reason for ICU admission or they may be seen in the setting of other types of brain injuries. Simple partial seizures manifest clinically as focal repetitive clonic movements without disturbance in consciousness, while complex-partial seizures are associated with disturbance in consciousness, and generalized tonic motor seizures, including those with secondary generalization, display early tonic rigidity followed by clonic convulsions [3]. Generalized seizures can be diagnosed clinically, but complex partial seizures are sometimes hard to diagnose, especially in patients with altered levels of consciousness. The other type of seizure in the ICU is NCSE, which requires EEG diagnosis [17]. Although NCSE is termed non-convulsive due to the paucity or absence of clinical signs, subtle clinical motor events may still be observed (Table 1) [3, 26]. Most NCSE seizures are documented within the first 24 h of EEG monitoring [10]. The prevalence of NCSE varies among clinical studies and it is seen in about 8 % of comatose patients [21]. Anoxia is the most common responsible etiology followed by traumatic brain injury (TBI), subarachnoid hemorrhage (SAH) and lobar intracranial hemorrhage (ICH) [21, 27]. When patients are unresponsive or when seizures are suspected to be responsible for the twitches, especially in brain injuries that are highly epileptogenic, EEG should be obtained and prolonged monitoring may be indicated (Fig. 1).

More recently, stimulus-induced seizures have become frequently recognized as subtle rhythmic or quasi-rhythmic twitching of the arm or limb that may last as long as the stimulus, which may include suctioning, loud noise or sternal rub [19]. EEG often shows epileptiform activity. In addition, EEG patterns of stimulus-induced rhythmic, periodic or ictal discharges (SIRPIDs) have been described in brain injury patients following various types of stimuli and may not be associated with clinical findings [28].

### Anoxic brain injury

Abnormal movements are common in the post-anoxic period [2]. When considering abnormal movements in this category, latency between the onset of the movement and anoxia is an important factor [2]. Seizures, acute post-hypoxic myoclonus (PHM) and shivering as a result of therapy itself (i.e., temperature control) dominate in the immediate post-anoxic period [29–31], while chronic PHM, dystonia, tremor and chorea appear in the chronic phase as patients become conscious [2].

Thus, an approach based on ruling out seizures in the acute period by conducting EEG or cEEG monitoring is the first step, especially since most patients are comatose (Fig. 1). In the chronic phase as patients become conscious, a different approach can be taken based on epidemiology and semiology of the observed movement. EEG should still be considered first when seizures are suspected.

The most well described abnormal movement following cardiac arrest is PHM [29]. PHM is considered acute when the myoclonus appears soon after cardiac arrest, typically within the first 24 h [2]. Status myoclonus is used when the movements last more than 30 min or they are repetitive and widespread involving both the face and limb and can be sound-sensitive or spontaneous [2, 32]. Acute PHM occurs most often in comatose patients, and manifests as a single or series of violent generalized myoclonic jerks that often involve the face [29]. The movements are difficult to control and are associated generally with a poor prognosis [1, 33]. The origin of acute PHM is still debated and an origin from the brain stem has been considered in light of the severity of cortical injury as usually noted in post-mortem studies [34].

In contrast, chronic PHM (Lance-Adams syndrome), dystonia and chorea emerge a few days to several weeks following anoxic injury, and rarely occur in comatose patients [2]. Chronic PHM has different semiology as it involves the limbs more often than the axial muscles and happens more on movements (intentional myoclonus), and a cortical origin of the movements has been proposed [2]. Dystonia, parkinsonism and akinetic rigidity occur secondary to basal ganglia injury because of hypoxia. They are often progressive, starting with involvement of the face and limbs [2, 35–37]. Chorea occurs less frequently than dystonia and it is different semiologically from the above as it involves rapid violent movements of the extremities compared with a paucity of movement in parkinsonism [37].

### Vascular brain injuries: acute ischemic stroke, ICH and SAH

Cerebrovascular events are frequently encountered in the ICU, and a wide variety of abnormal movements may result from the primary vascular injury or secondary injury that often follows due to edema, mass effect and disruption of the brain circuits [9, 15, 16, 38]. In the acute setting, spontaneous or stimulus-induced flexor or extensor posturing are often harbingers of compressive mass effect, which requires urgent intervention, so these should not be confused with seizures [9, 39].

Seizures are seen in 8.9 % of patients following stroke and are encountered more frequently in ICH than acute ischemic stroke (AIS) [16]. Forty percent of post-AIS seizures and 57 % of post-ICH seizures manifest within the first 24 h, accounting for a high proportion of abnormal movements during the acute period [16]. Abnormal movements are also common at the time of aneurysm rupture in SAH, although it is not clear whether such movements are seizures or posturing at the time of ictus [40]. Classic clinical seizures happen in only 1–7 % of SAH cases [40]. Thus, the approach should focus on ruling out either an epileptic origin or brain compression as the genesis of the abnormal motor phenomena, and serial brain imaging and EEG should be considered first priorities of investigation.

Other abnormal movements following stroke may include myoclonus, tremor, chorea, parkinsonism and hemiballism, and are together observed in 1–3.9 % of cases [15, 41]. These dyskinesias are often contralateral to the injured hemisphere [15]. Hemibalismus, chorea or hemichorea are the most common movement disorders following ischemic stroke [15, 41]. Hemibalismus commonly occurs following injury to the subthalamic nuclei [38] and is characterized by vigorous, irregular, high amplitude movements on one side of the body [42]. Of all paroxysmal movements, chorea, asterixis and hemibalismus have the shortest latency between stroke ictus and onset of movement and can be part of the acute stroke presentation [15, 43]. Differentiation of chorea from simple partial seizures may be challenging since EEGs may be uninformative in simple partial seizures [44]. The semiology of rapid disorganized movement that moves from one part of the body to another favors chorea, while repetitive synchronous movements, especially in crescendo fashion, favor a simple seizure.

The remainder of movements typically occur a few months after stroke and tend to resolve over time [42]. Dystonia is the second most common after chorea and neuroanatomically localizes to an injury to the putamen [42].

### Traumatic brain injury

Similar to cerebrovascular injuries, motor paroxysms such as posturing are related to cerebral herniation and extension of mass effect. Coupled with shivering and seizures, this triad of motor findings dominates the early period following TBI [45]. However, another source of abnormal movements in severe TBI is paroxysmal sympathetic hyperactivity, which happens in 15–33 % of comatose patients with severe TBI [46]. Paroxysmal sympathetic hyperactivity refers to cycling of dystonia, agitation, and decreased levels of consciousness combined with sympathetic symptoms of sweating, fever, tachycardia and hypertension. It may be seen within 24 h following trauma [46]. The diagnosis of this syndrome is made after ruling out other motor paroxysms related to cerebral herniation and seizures. Post-traumatic seizures (PTSs) are classified as early when they occur within the first 7 days, and late if they have their onset beyond this period [45]. Early PTSs happen in 4–25 % of TBI patients while the incidence of late PTSs in untreated patients varies between 9 and 42 % [45].

Other abnormal movements, including tremor, dystonia, tics, parkinsonism and chorea, may be seen several months following TBI in 13–66 % of cases and rarely in the acute period [47, 48]. Tremor followed by dystonia are the most common abnormal movements seen in TBI [47, 48].

### Toxic-metabolic brain injury

Various abnormal movements are frequently encountered in patients with toxic-metabolic brain injury. Severe metabolic derangements such as in liver failure, renal failure or hyponatremia, especially when acute in onset, may lead to cerebral edema and increased intracranial pressure (ICP) resulting in a comatose state with associated postural movements [49–51]. Seizures and NCSE may be seen as well in these severe cases [52]. Thus, we recommend serial brain imaging and EEG to rule out these emergencies, especially when the patient is unresponsive (Fig. 1). In addition, multiple nonspecific extrapyramidal movements may be encountered as well [53]. Asterixis or negative myoclonus is a nonspecific, frequent abnormal movement encountered in metabolic encephalopathy from liver disease, renal failure, respiratory failure, sepsis and cardiac disease [54]. It is characterized by irregular myoclonic lapses of posture affecting various parts of the body which are caused by 50–200 ms periods of relaxation of otherwise tonically active muscles [54]. In other situations, integrating the semiology of the movement with the etiological factor may lead to diagnosis in the awake patient or after ruling out seizures in the comatose patient (Fig. 1). For example, purposeless involuntary movements in the setting of non-ketotic hyperglycemia and associated magnetic resonance imaging (MRI) T1 hyperintensity of the putamen suggest chorea [55]. In a similar vein, the combination of irregular involuntary movements with twisting and writhing movements, known as choreoathetosis, happening in a paroxysmal or sustained fashion may be seen in the setting of hypoglycemia, hyperthyroidism or as a consequence of levothyroxine replacement therapy [56–58]. MRI may aid in the diagnosis in the case of hypoglycemia by showing scattered diffusion restriction lesions [56, 59]. Moreover, hyperexcitability of the nerves, primarily the facial nerve (Chvostek’s sign), resulting in temporal contraction of the muscle with stimulation, is seen in hypocalcemia, while parkinsonism and bilateral calcifications of the basal ganglia on head computed tomography scans may be seen in chronic hypocalcemia [60].

Similar to systemic illnesses, toxins and medications may cause an encephalopathic syndrome with abnormal movements. Patients may present initially to the ICU with one of these syndromes, such as neuroleptic malignant syndrome (NMS) [61, 62] or serotonin syndrome [63]. Rigidity is a common finding in NMS [13], and follows the initiation of neuroleptics and is usually associated with altered mental state, autonomic instability, fever, renal failure, and an elevation in serum creatine kinase [62]. Neuromuscular hyperactivity commonly occurs and manifests as rhythmic involuntary muscular contractions and relaxations (clonus), sudden brief muscular contractions (myoclonus) and tremor [63]. In addition, abnormal movement may also be a side effect of commonly administered ICU medications (Table 2) [58, 63–70]. A careful history, including a review of the timing and dosages of daily administered medications, should be conducted in all patients with newly observed movement. If possible, therapeutic withdrawal of a presumptive medication can serve as an important diagnostic and therapeutic intervention.

**Table 2:** Frequently used drugs in the intensive care unit associated with abnormal movements listed in alphabetical order

Drug class	Abnormal movement type with specific drug if applicable
Analgesics	Seizures (meperidine and tramadol) Clonus and myoclonus in the setting of serotonin syndrome (tramadol)
Antibiotics	Myoclonus and seizures with cefalosporins in renal failure Seizures (fluoroquinolones and carbapenems) Tremor (amphotericine) Clonus in the setting of serotonin syndrome (linezolid)
Antidepressants and mood stabilizers	Seizures (SSRI, TCA and bupropione) Tremor (lithium) Chorea (lithium)
Antiepileptics	Tremor (valproic acid) Ataxia and nystagmus (phenytoin) Seizures may be seen with toxic doses (phenytoin) Chorea (phenytoin, carbamazepine, valproic acid)
Antipsychotics	Seizures with almost all of them (especially clozapine) Acute dystonic reaction affecting mainly the cervical, cranial and axial muscles seen most commonly with typical neuroleptics such as haloperidol Neuroleptic malignant syndrome Akasthesia Tardive dyskinesia as a long-term effect Tremor
Cardiovascular agents	Seizures (digoxin toxicity) Tremor (amiodarone, procainamide)
Contrast agents	Seizures (diatrizoic acid)
Hormones	Tremor (levothyroxine, epinephrine) Chorea (levothyroxine)
Gastrointestinal agents	Acute dystonic reaction (metoclopramide) Neuroleptic malignant syndrome (metoclopramide) Tardive dyskinesia as a long-term effect (metoclopramide) Tremor (metoclopramide, cimetidine) Chorea (metoclopramide)
Misused drugs	Seizures (alcohol withdrawal, cocaine and amphetamine toxicity) Tremor (same drugs that cause seizures) Chorea (amphetamine, cocaine)

List of abnormal movements associated with each class of frequently administered medication in the intensive care unit setting. Examples refer to most frequently associated drug with abnormal movement type. *SSRI* selective serotonin reuptake inhibitor, *TCA* tricyclic antidepressant

### Infectious brain injury

Various abnormal movements may be seen in the setting of infections as a result of direct central nervous system invasion by the pathogen or indirect toxin or autoimmune effects [14, 70, 71]. Similar to other categories when presentation is acute, especially in comatose patients, we recommend serial brain imaging and EEG to investigate cerebral herniation and elevated ICP or seizures as the source of motor paroxysms (Fig. 1) [72]. However, after ruling out these emergencies or in awake patients, combining movement semiology with epidemiological factors may yield valuable information. Moreover, correct identification of the movement may point towards the etiological factor in certain scenarios. For example, opisthotonus posturing defined by severe muscle spasms causing backward arching of the head, neck and spine is typically seen in tetanus [73] and seizures are a common phenomenon with encephalitis or meningoencephalitis [74]. The presentation of complex partial seizures with encephalitic syndrome in a sporadic fashion suggests herpes simplex virus, which typically involves the temporal lobes [75], while encephalitis epidemic causing seizures suggests Japanese encephalitis, the most common epidemic viral cause of encephalitis [75]. In contrast, West Nile encephalitis is less commonly associated with seizures but may present with frequent extrapyramidal movements such as coarse tremor, myoclonus and parkinsonism [76]. Moreover, tuberculomas and tuberculosis meningitis are frequently associated with extrapyramidal movements, especially chorea and tremor [77, 78]. In addition, involuntary purposeless movement on one side of the body, known as hemichorea, is the most common extrapyramidal movement associated with AIDS [14]. On the other hand, rapidly progressive dementia associated with multifocal myoclonus is pathognomonic for Creutzfeldt-Jakob disease [79]. In addition, knowledge of endemic diseases or travel history may point to the movement etiology, such as seizures, ataxia and tremor in cerebral malaria [80] and seizures caused by neurocystcercosis [81]. In difficult cases, expert neurological consultation may be warranted to correctly identify the movement.

### Inflammatory brain injury

We refer in this section to diseases of primary autoimmune or paraneoplastic origin as opposed to inflammatory or immunological alterations accompanying other types of brain injuries [71, 82–85]. Encephalitidis associated with antibodies directed towards intracellular antigens (anti-Hu, anti-CRMP-5 or anti-Ma2) are often paraneoplastic while antibodies directed towards membrane antigens (anti-NMDA, anti-AMPA, anti-GABAb) are less commonly paraneoplastic [71]. In each case, a variety of abnormal movements may be seen [14, 71, 82, 83, 86].

Recognition of abnormal movements, disease presentation and underlying cancer if known may serve as powerful diagnostic tools for these syndromes [87, 88]. ICU admission may be required at presentation due to encephalopathy, coma, seizures or status epilepticus and the intensivist may face a challenging diagnosis [86]. Seizures are common in many of these diseases (Table 3) and EEG should be obtained if the patient’s mental state is altered or in case of clinical suspicion [86]. Moreover, other types of extrapyramidal movements may happen in isolation or in combination with seizures. For example, the combination of seizures with orofacial dyskinesia in the setting of ovarian teratoma highly suggests anti-NMDA encephalitis [71, 83]. Other associated neurological diseases may also aid in achieving the diagnosis, such as retinitis and neuropathy with CRMP5 antibodies [88, 89]. Spontaneous contractions affecting a small number of muscle fibers (fasciculations) or spontaneous muscular activity (neuromyotonia) may be seen with peripheral nervous system involvement, which may or may not accompany limbic encephalitis, as in the case of Morvan’s syndrome or Isaac’s syndrome, respectively [90].

**Table 3:** Commonly seen autoimmune antibodies, their related encephalitidis and associated abnormal movements listed in alphabetical order

Autoantibody	Clinical syndrome	Associated disease	Abnormal movement
Anti-amphiphysin	Encephalomyelitis	Breast cancer, SCLC	Myoclonus and seizures
Anti-CV2/CRMP-5	Encephalomyelitis, limbic encephalitis, retinitis and sensory neuropathy	SCLC, lymphoma	Seizures, chorea
Anti-Ri	Brain stem encephalitis	SCLC, breast cancer	Opsoclonus-myoclonus
Anti-NMDA	Limbic encephalitis	Ovarian teratoma	Seizures, orofacial dyskinesia, choreoathetosis, dystonia and combination of abnormal movements
Anti-AMPA	Limbic encephalitis	SCLC, thymoma	Seizures
Anti-GABA	Limbic encephalitis	SCLC	Seizures
Anti-TPO	Encephalitis	May be associated with thyroiditis	Seizures, myoclonus, tremor and chorea
Antiphospholipid antibodies	Encephalitis, strokes	Primary or in the setting of SLE	Seizures, chorea

Paraneoplastic antibodies, their associated tumors, clinical syndromes and abnormal movements. *AMPA* α-amino-3-hydroxy-5-methyl-4-isoxazolepropionic acid, *CRMP-5* collapsin response mediator protein, *GABA* gamma aminobutyric acid, *NMDA* N-methyl-D-aspartate, *SCLC* small cell lung cancer, *SLE* systemic lupus erythematosus, *TPO* thyroid peroxidase

On the other hand, Sydenham chorea comprises autoimmune sequelae that happen a few months after streptococcal infection in children [71]. Hashimoto’s thyroid disease with anti-TPO antibodies may be associated with unresponsiveness, myoclonus and seizures [91]. This entity is very responsive to steroids and a trial of steroids may serve both diagnostic and therapeutic purposes [91]. Other diagnostic modalities, such as brain MRI and cerebrospinal fluid analysis, showing typical inflammatory changes and identifying the paraneoplastic antibody may aid in the diagnosis. Expert neurological consultation may be necessary in unusual cases (Fig. 1).

### Brain tumors

Primary and metastatic brain tumor patients may experience cerebral edema with associated herniation and elevation of ICP resulting in paroxysmal posturing [92, 93]. Abnormal movements related to seizures are seen in 60 % of primary brain tumors and 49 % of metastatic brain tumors [93]. Low-grade tumors tend to be more epileptogenic than high-grade tumors [93]. Seizures in these cases are usually focal with or without secondary generalization, or they may be complex partial, suggestive of focal onset [93]. Simple partial continua with continuous shaking of a limb or the face may also be seen [94].

In contrast to other types of brain injuries, tumors are rarely responsible for extrapyramidal movements [95]. Intrinsic basal ganglia and thalamus tumors have been reported to cause chorea and less commonly dystonia [95].

## Conclusion

Abnormal movement is an important phenomenon in the ICU and is associated with diverse etiologies and therapeutic implications. Based on previous data, a high degree of vigilance in identifying seizures and motor paroxysms due to impending herniation should be maintained, as early intervention often leads to improved outcome [96, 97]. Although not urgent, identification of other abnormal movements should not be overlooked and may prove very helpful in ICU patient management. An approach based on integrating the semiology and frequency of the movement according to the brain injury type may lead to correct diagnosis. Expert neurological consultation may be necessary in difficult cases.

## Abbreviations

AIS: Acute ischemic stroke
cEEG: Continuous electroencephalogram
EEG: Electroencephalogram
ICH: Intracranial hemorrhage
ICP: Intracranial pressure
ICU: Intensive care unit
MRI: Magnetic resonance imaging
NCSE: Non-convulsive status epilepticus
NMS: Neuroleptic malignant syndrome
PHM: Post-hypoxic myoclonus
PTS: Post-traumatic seizure
SAH: Subarachnoid hemorrhage
SIRPID: Stimulus-induced rhythmic periodic or ictal discharge
TBI: Traumatic brain injury
